# A subset of low density granulocytes is associated with vascular calcification in chronic kidney disease patients

**DOI:** 10.1038/s41598-019-49429-x

**Published:** 2019-09-13

**Authors:** Javier Rodríguez-Carrio, Natalia Carrillo-López, Catalina Ulloa, Mariana Seijo, Minerva Rodríguez-García, Carmen Rodríguez-Suárez, Carmen Díaz-Corte, Jorge B. Cannata-Andía, Ana Suárez, Adriana S. Dusso

**Affiliations:** 1 Area of Immunology, Department of Functional Biology, University of Oviedo, Oviedo, Spain; 2 Bone and Mineral Research Unit, Hospital Universitario Central de Asturias, Instituto de Investigación Sanitaria del Principado de Asturias (ISPA), Oviedo, Spain; 3 Red de Investigación Renal (REDinREN), Instituto de Salud Carlos III (ISCIII), Hospital Universitario Central de Asturias, Oviedo, Spain; 4 Division of Nephrology, Hospital Universitario Central de Asturias, Oviedo, Spain; 5 Laboratorio de Enfermedades Metabólicas Óseas, Hospital de Clínicas, Instituto de Inmunología, Genética y Metabolismo (INIGEM) CONICET- UBA, Buenos Aires, Argentina; 6 Department of Medicine, University of Oviedo, Oviedo, Spain

**Keywords:** Diagnostic markers, Translational research, Kidney diseases

## Abstract

Inflammation is central to chronic kidney disease (CKD) pathogenesis and vascular outcomes, but the exact players remain unidentified. Since low density granulocytes (LDGs) are emerging mediators in inflammatory conditions, we aimed to evaluate whether LDGs may be altered in CKD and related to clinical outcomes as biomarkers. To his end, LDGs subsets were measured in peripheral blood by flow cytometry and confocal microscopy in 33 CKD patients undergoing peritoneal dialysis and 15 healthy controls (HC). Analyses were replicated in an additional cohort. DEF3 (marker of early granulopoiesis) gene expression on PBMCs was quantified by qPCR. Total CD15^+^ LDGs and both CD14^low^CD16^+^ and CD14^−^CD16^−^ subsets were expanded in CKD. The relative frequency of the CD14^−^CD16^−^ subpopulation was higher among the CD15^+^ pool in CKD. This alteration was stable over-time. The increased CD14^−^CD16^−^CD15^+^ paralleled Kauppila scores and DEF3 expression, whereas no association was found with CD14^low^CD16^+^ CD15^+^. Both subsets differed in their CD11b, CD10, CD35, CD31, CD62L, IFNAR1 and CD68 expression, FSC/SSC features and nuclear morphology, pointing to different origins and maturation status. In conclusion, LDGs were expanded in CKD showing a skewed distribution towards a CD14^−^CD16^−^CD15^+^ enrichment, in association with vascular calcification. DEF3 expression in PBMC can be a marker of LDG expansion.

## Introduction

Chronic Kidney Disease (CKD) is a disorder characterized by premature and exacerbated multi-organic aging^1^. As a consequence, CKD patients develop a number of senescence-related clinical outcomes, such as atherosclerosis, osteoporosis, soft tissue calcifications, sarcopenia, frailty, infections, oxidative stress, etc. Importantly, vascular calcification (VC) in CKD patients is a main determinant of their increased risk of cardiovascular (CV) death compared to the general population^2–4^.

Chronic and dysregulated inflammation plays a pivotal role in CKD progression, although the exact inflammatory mediators remain unclear at present. Inflammatory pathways are key factors for VC^5,6^, an important hallmark of CKD. Of note, inflammation is considered a major part of the aging process and recent studies have brought to light its involvement in CV outcomes^7,8^. Most vascular risks in CKD seem to be attributed to medial calcification rather than atherosclerosis occurrence (reviewed in^9^). However, immune circuits associated with VC in CKD are poorly understood. Unravelling immune mediators that underlie VC in CKD is of upmost relevance both from the basic perspective as well as for the clinical translation of such findings. In this scenario, myeloid populations have been partially neglected in CKD and VC. Importantly, novel aspects of granulocyte biology have emerged in recent years.

A major novel breakthrough in the field was the discovery of the enormous heterogeneity among granulocytes^10,11^. Additionally, granulocytes are now recognized as immune cells that can perform complex activities, orchestrate the immune response via several mediators and cytokines and establish a complex crosstalk with components of the innate and adaptive response^12,13^. In this scenario, a novel subset of granulocytes, the low density granulocytes (LDGs) are emerging as relevant players in a wide range of immune-based conditions^14–17^. LDGs are defined by their ability to sediment in the PBMC fractions upon gradient centrifugation of whole blood and to exhibit granulocyte markers. Nevertheless, a precise phenotypic definition of LDGs is lacking. LDGs have received a notable attention since their frequency has been related to disease severity and clinical outcomes in a number of conditions (reviewed in^17^). Among these conditions, systemic lupus erythematosus has been hallmarked by a noticeable LDG expansion^17,18^. Interestingly, a recent study has found an upregulation of a granulocyte-related gene, the defensing 3 (DEF3A) in PBMC isolates from patients^19^. Although these lines of evidence may suggest that DEF3A could be a promising candidate of LDGs expansion, this potential connection has not been studied.

However, whether LDGs may be involved in CKD outcomes is yet to be clarified. Therefore, in the present study we aimed (i) to evaluate LDGs frequency in CKD patients, (ii) to analyze the associations between LDGs and clinical features in this condition as well as their role as a biomarker and (iii) to assess the LDGs phenotype in CKD.

## Results

### LDG expansion in CKD patients

**Figure 1:**
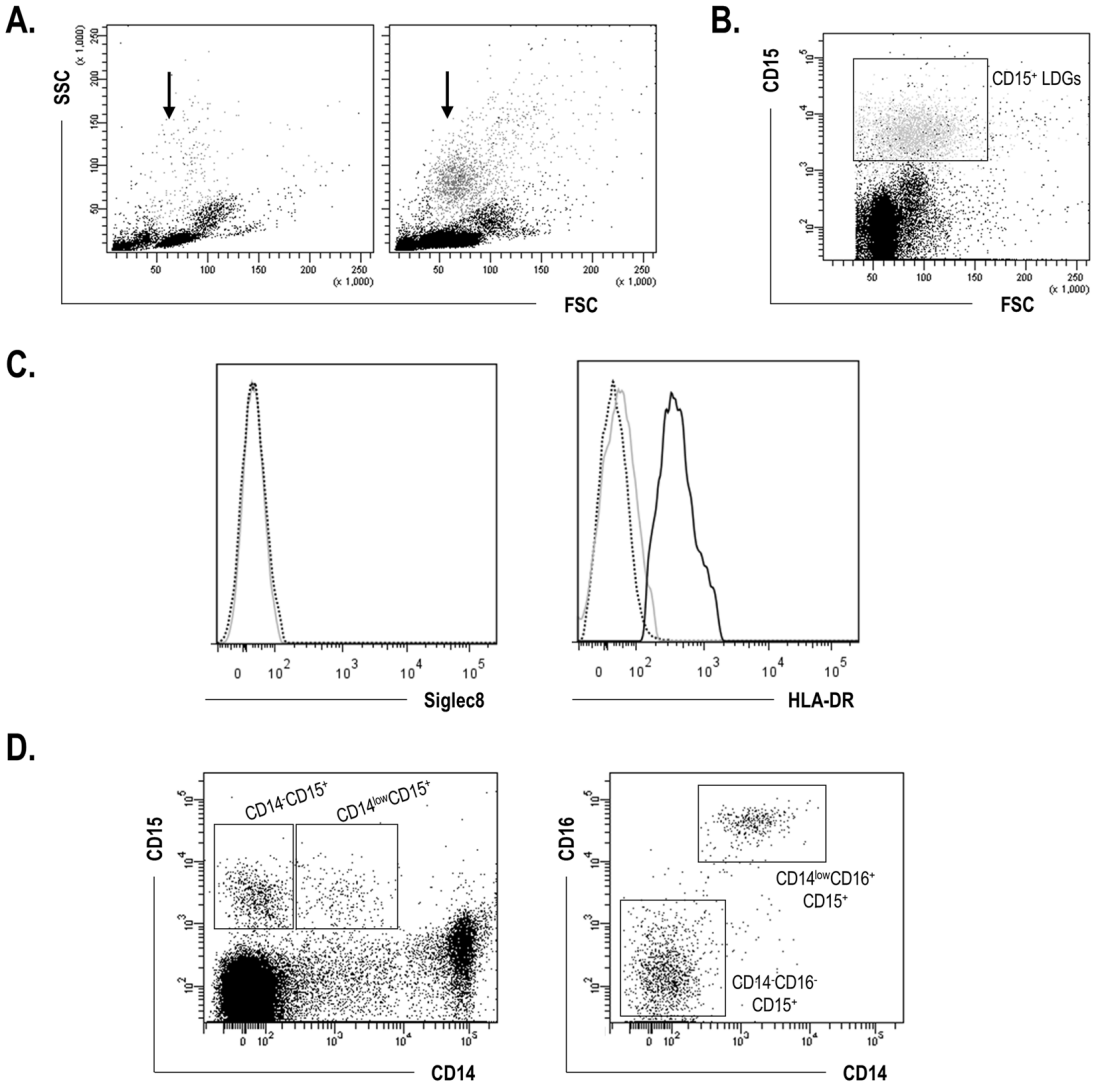
Gating strategy for LDG identification and quantification. (**A**) LDG (arrows) were identified by their FSC/SSC properties in PBMC fractions. Representative dot plots from a HC (left) and a CKD5-PD patient (right) are shown. (**B**) LDGs were first identified by their CD15 expression. (**C**) Histograms showing the analyses of the expression of Siglec8 (isotype control: dotted black line, CD15^+^ population: gray line) and HLA-DR (isotype control: dotted black line, CD15^+^ population: gray line, monocytes: black line). (**D**) Two subsets of LDGs were distinguished within the total CD15^+^ population based on their CD14 (left dot-plot) and CD16 (right dot-plot) expression: CD14^−^CD6^−^CD15^+^ and CD14^low^CD16^+^CD15^+^.

The presence of LDGs was evaluated in a group of 33 CKD5-PD patients and 15 HC (Table 1). The LDG population clearly segregated from the monocytes and lymphocytes subsets within the PBMC fraction by its side scatter signal (Fig. 1A). LDGs also clearly differed from monocytes by the expression of HLA-DR, their FCS/SSC signal as well as by the granulocyte marker CD15 (Fig. 1B,C). Additionally, CD15^+^ cells were negative for Siglec8 expression (Fig. 1C), thus ruling out the possibility of these cells to be eosinophils. Interestingly, two different subsets were observed when the CD14 expression was analyzed, in a similar way than that of CD16. As a consequence, two subpopulations could be distinguished within the CD15^+^ LDG population: CD14^−^CD16^−^CD15^+^ and CD14^low^CD16^+^ CD15^+^ (Fig. 1D).

**Table 1:** Demographical, clinical and immunological characteristics of individuals recruited for this study.

	HC (n = 15)	CKD5-PD patients (n = 33)	p-value
Age, years, mean (range)	48.00 (22.00–68)	55.00 (21.00–77.00)	0.070
Gender, f/m	10/5	13/20	0.080
***Clinical features***			
Albumin, mg/dl	44.17 ± 1.95	34.37 ± 4.35	<0.001
Urea, mg/dl	33.46 ± 8.08	132.66 ± 43.76	<0.001
Creatinine, mg/dl	0.78 ± 0.17	7.81 ± 2.72	<0.001
Plasma Ca, mmol/l	2.34 ± 0.07	2.17 ± 0.16	<0.001
Plasma phosphate, mmol/l	1.04 ± 0.18	1.64 ± 0.42	<0.001
PTH, pg/ml	49.60 ± 15.52	371.03 ± 198.61	<0.001
25(OD)-vitamin D, ng/ml	32.93 ± 15.38	10.10 ± 6.89	<0.001
CRP, mg/dl		0.64 ± 0.86	
Vascular calcifications, n(%)		18 (54.5)	
Kauppila score		7.88 ± 8.98	
Time on dialysis, months		15.00 (16.00)	
***Treatments, n(%)***			
Paricalcitol		15 (45.4)	
Phosphate binders		18 (54.5)	
Statins		21 (63.6)	
Metilprednisone		9 (27.22)	
Epo		24 (72.7)	
***Serum cytokines, pg/ml***			
IL-2	1.30 (1.91)	1.60 (0.70)	0.342
IL-6	4.73 (4.58)	6.77 (10.07)	0.154
IL-10	1.49 (0.80)	1.77 (1.05)	0.101
IFNγ	8.32 (6.80)	12.82 (6.57)	0.183
TNFα	8.38 (7.66)	14.01 (6.38)	0.211

**Figure 2:**
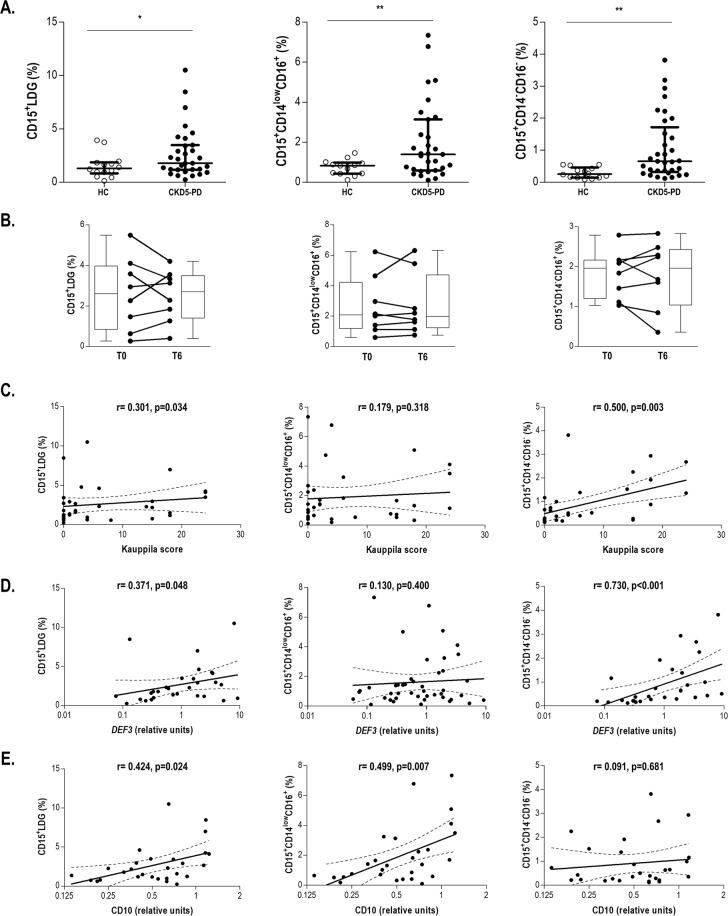
LDG subsets in CKD5-PD patients and their association with clinical features. (**A**) The frequencies of total CD15^+^ LDGs as well as CD14^−^CD6^−^CD15^+^ and CD14^low^CD16posCD15^+^ subsets in PBMC fractions were compared between HC (open circles) and CKD5-PD patients (black circles). Central bars represent the median values whereas whiskers represent 25^th^ and 75^th^ percentiles. Differences between groups were assessed by Mann-Withney U tests (*p < 0.050, **p < 0.010, ***p < 0.001). (**B**) Analyses of LDG subsets in paired samples (n = 8) at baseline (t = 0) and after 6 months (t = 6). LDG frequencies are expressed as bars and scatter plots for each time point. Differences were assessed by Wilcoxon test for paired samples. Association of the LDG subsets with Kauppila scores (**C**), DEF3 (**D**) and CD10 (**E**) gene expression in PBMC fractions. Correlations were assessed by Spearman’s rank test.

CKD5-PD patients exhibited a higher frequency of LDGs within the mononuclear fraction than HC, and higher levels were observed for both CD14^low^CD16^+^ CD15^+^ and CD14^−^CD16^−^CD15^+^ subsets (Fig. 2A). None of the LDG subsets were related to age, time on dialysis, medications or circulating cytokines (all p > 0.050). Moreover, no differences in circulating neutrophils were observed between patients and controls and none of the LDG subsets were correlated to neutrophil absolute counts (all p > 0.050). Interestingly, within the CD15^+^ total population, the relative frequency of the CD14^−^CD16^−^ subset was increased in patients compared to HC (24.59 ± 19.50 vs. 12.21 ± 10.12, p = 0.012), thus pointing to a skewed profile of the LDG pool in CKD. Additionally, a subgroup of patients (n = 8) was re-sampled after 6 months, and LDGs frequency and their relative distribution in the LDG pool were maintained over this period (Fig. 2B).

**Figure 3:**
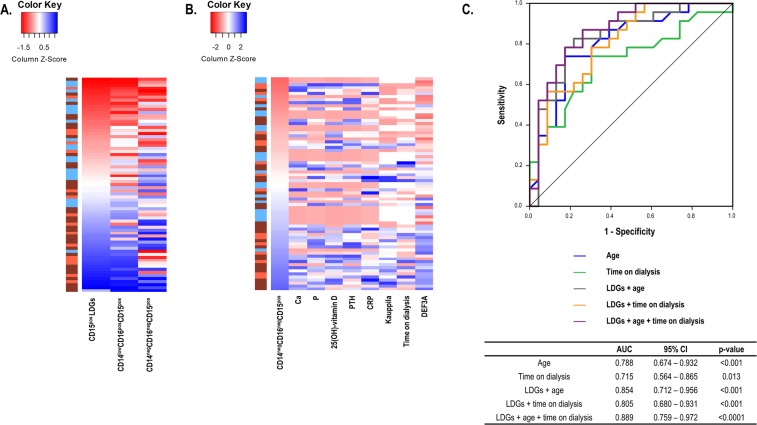
LDG subsets as biomarkers. (**A**,**B**) Associations of the frequency of the CD14^−^CD16^−^CD15^+^ population with other LDG subsets and clinical features. Variables were plotted in heatmaps (**A**, ranked by CD15^+^ LDG frequency; B, ranked by CD14^−^CD16^−^CD15^+^ frequency). Each row of the heatmaps represents a study subject. Colors in the vertical left bar denoted HC (blue), CKD5-PD patients (brown) or CKD5-HD patients (orange). Tiles are colored based on variable levels, red and blue indicating low or high levels respectively, as indicated in the legend (top left). (**C**) ROC analyses of the role of CD14^−^CD16^−^CD15^+^ as a biomarker to predict VC. ROC curves were plotted and AUC, 95% CI and p-values were computed (table).

Interestingly, CD14^−^CD16^−^CD15^+^ expansion was restricted to patients with vascular calcifications (VC, Kauppila scores > 5) compared to VC-free patients (1.29 ± 1.09 vs. 0.41 ± 0.33%, p = 0.010) and HC (0.34 ± 0.26%, p = 0.009), whereas this effect was not observed in the CD14^low^CD16^+^ CD15^+^ subpopulation (p = 0.245 and p = 0.145, respectively). In fact, the frequency of CD14^−^CD16^−^CD15^+^ LDGs was strongly correlated to Kauppila scores in the whole patient group (Fig. 2C). Importantly, whereas the CD14^low^CD16^+^ CD15^+^ frequency strongly mirrored that of total CD15^+^ LDGs, this was not the case for CD14^−^CD16^−^CD15^+^, which exhibited a different distribution (Fig. 3A,B).

Furthermore, to gain insight into the LDG expansion, the gene expression of DEF3 (a granulocyte-restricted gene among blood cells) was measured in PBMC fractions. CKD5-PD patients exhibited a higher DEF3 expression compared to HC, which did not reach statistical significance (1.02(2.56) vs. 0.55(0.76) relative units, p = 0.128). Surprisingly, DEF3 expression was strongly correlated with the CD14^−^CD16^−^CD15^+^ subset but not with the CD14^low^CD16^+^ CD15^+^ subpopulation (Fig. 2D). DEF3A expression was in turn associated with the Kauppila score (r = 0.501, p = 0.006).

Similarly, the gene expression of CD10 (a well-known marker of mature neutrophils) was analyzed in PBMC fractions. CD10 expression paralleled total CD15^+^ LDGs levels (Fig. 2E). However, this correlation was observed to be driven by the CD14^low^CD16^+^ CD15^+^ subset, whereas no association was found with their CD14^−^CD16^−^CD15^+^ counterparts.

Finally, a replication cohort consisting on 16 CKD patients undergoing hemodialysis (CKD-5HD) and 6 age- and gender-matched controls were recruited to validate our findings. The independent analysis of this cohort confirmed all of our previous results (Supplementary Fig. 1). No differences were observed between dialysis modalities.

The ability of CD14^−^CD16^−^CD15^+^ LDGs as a biomarker to identify VC was tested by ROC curves. Interestingly, an AUC [95% CI] of 0.769 [0.613, 0.924] (p = 0.006) was obtained for the CKD5-PD cohort. This was confirmed in the CKD5-HD group (0.803 [0.622, 0.984], p = 0.014). A pooled analysis confirmed a good discriminative power of CD14^−^CD16^−^CD15^+^ LDGs as a biomarker in CKD (Fig. 3C). Moreover, not only it was identified as an independent predictor but also the frequency of CD14^−^CD16^−^CD15^+^ LDGs improved the ability of age and time on dialysis to predict VC when included in combined indices (Fig. 3C). Finally, multiple logistic regression analysis confirmed that CD14^−^CD16^−^CD15^+^ LDGs were independent predictors of VC (OR [95% CI], p: 1.112 [1.012–9.175], p = 0.020) after adjusting for age, gender and time on dialysis as confounders.

Taken together, these results suggest that CKD is hallmarked by a systemic LDG expansion that is stable over time. LDGs in CKD patients are a heterogeneous population, the CD14^−^CD16^−^CD15^+^ subset being associated with calcification, thus supporting its role as an independent biomarker. DEF3 expression in the PBMC fraction, which parallels CD14^−^CD16^−^CD15^+^ levels, could be considered as a surrogate marker of CD14^−^CD16^−^CD15^+^ levels and thus, a biomarker of VC.

### Resolving the LDG heterogeneity in CKD

In order to gain insight into the heterogeneity observed within the LDG pool in CKD, an extensive immune-phenotyping of these populations and of mature neutrophils was carried out.

**Figure 4:**
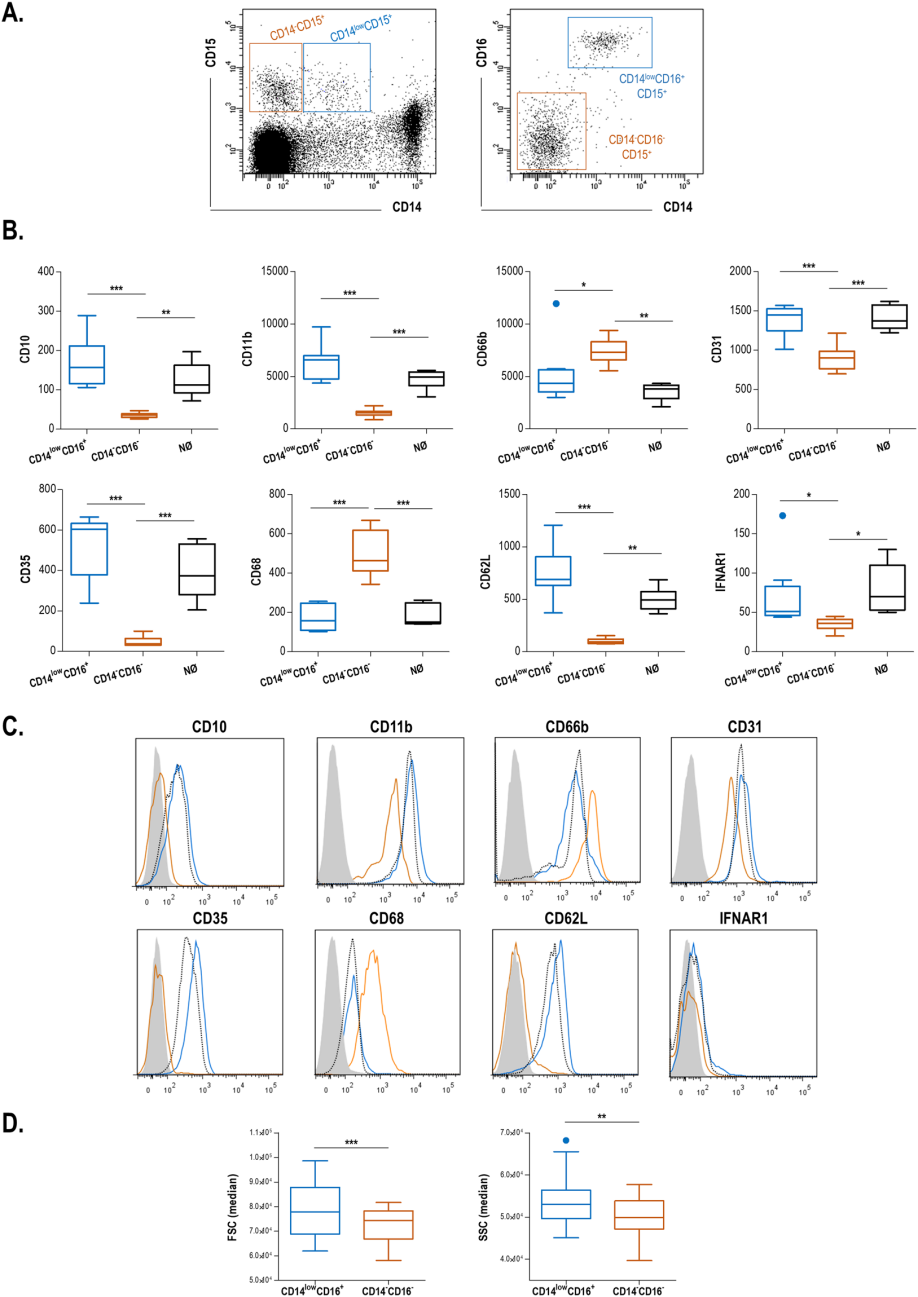
Phenotype of LDG subsets in CKD. (**A**) CD14^−^CD6^−^CD15^+^ (orange) and CD14^low^CD16posCD15^+^ (blue) subsets were selected based on the previous gating strategy. (**B**) The expression of several granulocyte markers was analyzed by flow cytometry and compared among LDG subsets and mature neutrophils (black). Expression levels are shown as box plots, where the boxes represent the 25th and 75th percentiles, the lines within the boxes representing the median and the lines outside the boxes represent the minimum and maximum values. Differences were assessed by Kruskal-Wallis tests with Dunn-Bonferroni correction for multiple comparisons’ test. (**C**) Representative histograms for granulocyte markers (gray: isotype control, orange: CD14^−^CD6^−^CD15^+^ subset, blue: CD14^low^CD16posCD15^+^ subset and black: mature neutrophils). (**D**) Differences in FSC and SSC parameters between both LDG subsets. Expression levels are shown as box plots, where the boxes represent the 25th and 75th percentiles, the lines within the boxes representing the median and the lines outside the boxes represent the minimum and maximum values. Differences were assessed by Mann-Withney U tests or paired tests (Wilcoxon’s test). *p < 0.050, **p < 0.010, ***p < 0.001.

Interestingly, the CD14^low^CD16^+^ CD15^+^ subset exhibited an increased expression of mature and activated granulocyte markers (CD11b, CD10, CD35, CD31, CD62L and IFNAR1), whereas the CD14^−^CD16^−^CD15^+^ subpopulation exhibited a higher expression of CD66b and CD68, the latter being a marker of intermediate and early stages of neutrophil differentiation (Fig. 4A,B). As a consequence, the CD14^low^CD16^+^ CD15^+^ subset exhibited a profile that resemble that of mature neutrophils (Fig. 4C), whereas a distinct one was observed for their CD14^−^CD16^−^CD15^+^ counterparts. Additionally, these two subsets also differed in their size (FSC) and granularity (SSC) features (Fig. 4D).

**Figure 5:**
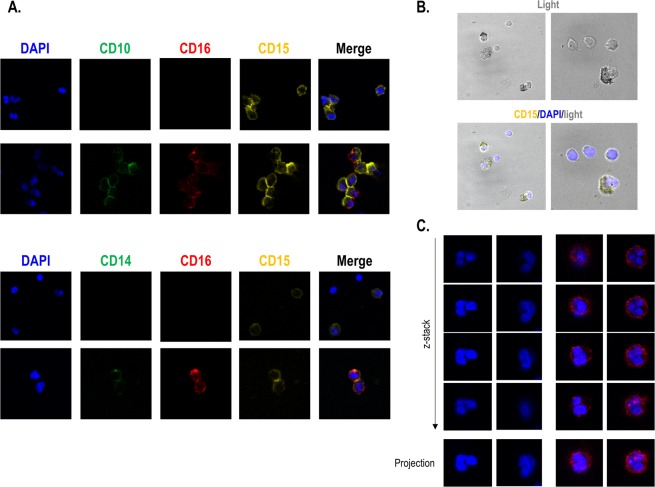
Microscopy analyses of LDG subsets. (**A**) Enriched CD15^+^ cells from PBMC isolates were stained with CD15 (PE), CD16 (APC) and CD14 or CD10 (FITC) and nuclei were counterstained with DAPI. Surface expression of the markers allowed the identification of the two subsets. Four representative images are shown. (**B**) Cell morphology was analyzed by transmitted light, in combination with fluorescence channels. Two fields where lymphocytes could be observed were chosen to allow comparison between LDG and lymphocytes regarding cell size and complexity, cytoplasm distribution and nuclear morphology. Two representative images are shown. (**C**) Nuclear morphology was very heterogeneous between LDG subsets. Those CD16^+^ mostly exhibited a polymorphonuclear shape, whereas a lower degree of nuclear segmentation was observed for those CD16^−^. Z-stacks were used to visualize the nuclei complexity among different sections, which evidenced the existence of different lobules, if present. Two representative images are shown. Original magnification X630 for all the images.

The phenotype of the LDGs was also confirmed by fluorescence confocal microscopy (Fig. 5). In CD15^+^ enriched fractions, both CD14^low^CD16^+^ CD15^+^ and CD14^−^CD16^−^CD15^+^ subsets were identified, CD14/CD10 and CD16 co-expression being confirmed in the former (Fig. 5A). Interestingly, CD14 expression was observed to be lower than other markers, such as CD10, in accordance to the CD14^low^ expression detected by flow cytometry. The analysis of cell morphology (Fig. 5B) confirmed that LDGs were bigger than lymphocytes (as revealed by flow cytometry) and exhibited a complex membrane, in accordance with the SSC signal. Differences in the nucleus were also evident between LDGs and lymphocytes. Furthermore, examination of the nuclear morphology by DAPI counterstaining confirmed the complex structure of the LDGs nuclei (Fig. 5C). Importantly, different nuclear morphologies could be distinguished: CD16^+^ LDGs were more likely to exhibit a polymorphonuclear morphology, whereas those lacking CD16 expression exhibited a less segmented profile (bi-lobular, indented or band-like).

In conclusion, our results confirm that the LDG pool in CKD is composed by two distinct populations, CD14^low^CD16^+^ CD15^+^ showing a mature neutrophil-like phenotype, whereas CD14^−^CD16^−^CD15^+^ exhibited a distinct, immature profile. Nuclear morphology was also different between LDG subsets, which may account, at least in part, for the differences in the SSC signal.

## Discussion

Chronic inflammation, exacerbated aging and organ damage are well recognized hallmarks of CKD. Despite considerable advances striving to identify the exact immune cell subsets involved, a number of knowledge gaps are still present. Over the last decade, the study of innate immunity in CKD, and especially of granulocytes, has been partially neglected. However, in recent years an emerging body of evidence has highlighted a pivotal role for LDGs in a wide range of conditions, although, their relevance in CKD had not been studied. Herein, we have characterized for the first time the LDG pool in CKD, with a focus on its phenotype, heterogeneity and its clinical relevance as a potential biomarker. Moreover, this study provides a proof-of-concept of the significance of the granulocyte gene DEF3 as a surrogate marker of LDG expansion and thus, of vascular calcification in CKD.

LDGs have gained notable attention because of its pro-inflammatory properties and their particular characteristics^16,17^. However, their origin and phenotype are far from being clear. Actually, there is a notable heterogeneity in the scientific literature about the LDG identification^17^). A consensus strategy for LDG identification is lacking, and several markers and phenotypes have been proposed, which limits the comparison among studies. Despite of the huge variation in the markers proposed among studies, LDGs are usually conceived as a uniform population in individual studies, rather than a complex pool. Our results not only suggest that two phenotypically distinct LDG subsets can be distinguished but also, that they differ in terms of their clinical relevance. However, whether this finding could be applied to other clinical scenarios remains unknown, since the literature points to divergent functions of the LDG expansion in different scenarios, hence adding another layer of complexity to this field^17,20,21^. LDGs exhibiting CD14^low^ and CD16 expression were hallmarked by their elevated expression of mature and terminally-differentiated granulocytes and molecules of adhesion, as well as by their higher size and granularity compared to their CD14/CD16negative counterparts. Interestingly, CD14^low^CD16^+^ LDGs exhibited a high expression of CD10, which mirrored that of neutrophils, whereas it was absent in their CD14^−^CD16^−^ counterparts. Importantly, CD10 is specifically expressed by mature neutrophils at their latest stages of differentiation^22–24^, thus pointing to differences in maturation status between both LDGs subsets. Moreover, CD10 has been previously found to resolve heterogeneity among granulocytes in patients with acute or chronic inflammatory conditions in terms of their maturation status, hence strengthening our findings^25^. Moreover, CD14^low^CD16^+^ LDGs were hallmarked by a high expression of CD35, another well-known marker of mature neutrophils^22,26^ that is expressed in the plasma membrane after being shed from the secretory granules^27^, hence not only confirming the late maturation of this LDG subset but also suggesting a link with neutrophil degranulation. On the other hand, CD16^−^CD14^−^ LDGs showed a higher expression of CD68, a marker of the early stage of neutrophil differentiation^28^, and exhibited a strong correlation with DEF3 expression, a well-known marker of early granulopoiesis^19,28,29^. The opposed distribution of CD35 and DEF3A aligns with the different content of neutrophil granules along their differentiation stages^30,31^. Taken together, all these lines of evidence lead us to hypothesize that LDGs in CKD have different origins: whereas the CD14^low^CD16^+^ CD15^+^ cells are likely to be mature degranulated neutrophils, their CD14^−^CD16^−^CD15^+^ counterparts may represent a distinct, immature subpopulation of granulocytes prematurely released from the bone marrow. The differences observed for size and granularity are in line with this notion. Furthermore, our findings are in line with a different pattern of mobilization between these two subsets (Fig. 3), hence strengthening these notions. Surprisingly, a recent paper by Sagiv and coworkers revealed no differences in the number of granules between low density granulocytes and mature neutrophils in cancer patients^32^, which may contradict, at least in part, our hypothesis. However, due to the phenotypic and functional differences of LDGs in cancer and chronic inflammatory conditions, these results must be interpreted with caution. Additionally, differences in granularity could be also attributed, at least in part, to nuclear morphology and plasma membrane complexity. The microscopy findings observed in this study are in agreement with this idea.

Despite being less abundant than other leukocytes and exhibiting an immature phenotype, LDGs have been reported to prompt innate immune mechanisms, secrete pro-inflammatory cytokines, produce reactive oxygen species and, more importantly, to form Neutrophil Extracellular Traps (NETs)^18,33,34^. In fact, their immature state has been related to genetic damage and genomic instability in lupus patients^35^, which is supposed to underlie its aberrant functionality. Therefore, LDGs are likely to perpetuate chronic inflammation and tissue damage, two prominent features of CKD pathogenesis. LDGs exhibit an enhanced production of pro-inflammatory cytokines such as TNFα, IL-17 or IFNγ^18,33,34^. These mediators have been described to be related to CKD (immuno)-pathogenesis^36–38^. Interestingly, LDGs have been reported to impair vascular repair and promote direct endothelial damage^18,33,39^. Therefore, based on the literature, LDGs may be conceived as promising mediators of VC and multi-organ aging in CKD, thus warrantying further mechanistic studies in the future.

Interestingly, a recent paper has brought to light the existence of a network of trans-cortical capillaries in long bones that form a direct connection between the endosteal and periosteal circulations^40^. These vessels effectively transport blood and express endothelial markers that can guide neutrophil trafficking to the peripheral compartment. The number of these vessels as well as their activation status was determined by increases in TNFα expression and osteoclast activity, and autoimmune diseases affecting bone physiology led to substantial changes in transcortical vessel number^40^. Therefore, it may be hypothesized that increased trans-cortical capillaries formation could account, at least in part, for the increased frequency of LDGs in peripheral blood in CKD. However, whether trans-cortical capillaries are increased in CKD remains to be clarified.

A remarkable finding from our study was the association between CD14^−^CD16^−^CD15^+^ frequency and DEF3 expression in PBMC fractions. On one hand, DEF3 is a transcript restricted to the granulocyte lineage and as such, it should not be detected in PBMC fractions. Although this was the case for HC, it does not hold true for CKD patients since a significant proportion showed a notable upregulation of this gene. Therefore, an increased DEF3 expression in the PBMC fractions can be attributed to a LDG expansion, as demonstrated by our findings. On the other hand, DEF3 is only expressed during the intermediate (myelocyte-metamyelocyte) stage of neutrophil maturation^28,29^. The fact that the DEF3 expression was correlated with CD14^−^CD16^−^CD15^+^ frequency, but not with that of CD14^low^CD16^+^ CD15^+^ cells, confirms the immature status of the former and further supports the differences between these two subsets revealed by the immune phenotype performed. Taken together, our findings highlight a potential role of DEF3 expression as a marker of immature LDGs expansion. Although interesting from the mechanistic point of view, LDG quantification could be accompanied by technical and logistical limitations regarding its implementation in the clinical setting, as it requires the access to flow cytometry and handling cell biomarkers. However, the analysis of the DEF3 expression will have several advantages as a surrogate marker of LDG expansion and consequently, of the presence of VC. Interestingly, DEF3 has been linked to a number of adverse vascular outcomes (dyslipidemias, vascular and endothelial dysfunction, cardiovascular morbidity and mortality)^41–44^, hence reinforcing this point. Unfortunately, there is a profound knowledge gap about its functional involvement in the setting of these conditions.

LDG subsets were found to be stable over time. More importantly, LDGs were expanded to a similar extent in CKD patients undergoing peritoneal dialysis and hemodialysis, despite exhibiting a different time on dialysis. These results may suggest that LDG expansion is an early phenomenon and not the result of the disease progression itself. However, the possibility that LDGs expansion may be a consequence of the inflammatory condition associated with CKD and/or an epiphenomenon of the dialysis itself should not be ruled out. This represents a limitation of the present study that must be acknowledge. Moreover, the lack of functional assays does not allow us to evaluate the potential contribution (and differences) of the LDGs subsets to CKD pathogenesis. Further studies, with different patient populations and complementary methods are needed. Due to the heterogeneity of LDGs functional assays and the long-lasting for time frames of the clinical endpoints analyzed, a special focus must be considered at this stage to make a conscientious selection of functional assays and clinical endpoints.

In conclusion, the results herein presented suggest for the first time the alterations of LDGs in CKD. The LDG pool in CKD patients was more complex than initially conceived, with two subsets being identified and differing in their phenotype, nuclear morphology and presumably origin. Moreover, our study points to immature LDG as potential biomarkers of VC and sheds new light into the potential role of DEF3 expression as a relevant surrogate marker of LDG expansion in CKD. Our findings set the bases to design further functional and prospective studies to gain additional insight into this topic.

## Material and Methods

### Ethics statement

Approval for the study was obtained from the Institutional Review Board (Comité de Ética Regional de Investigación Clínica, reference PI16/00113), in compliance with the Declaration of Helsinki. All participants gave a written informed consent prior to their inclusion in the study.

### Patients and controls

Our study involved 33 CKD patients on peritoneal dialysis (CKD5-PD) recruited from the Peritoneal Dialysis Outpatient Clinic (Unidad de Gestión Clínica de Nefrología) at the Hospital Universitario Central de Asturias (HUCA, Oviedo, Spain). Simultaneously, a group of 15 healthy volunteers from the general population was recruited as healthy controls (HC). Moreover, 16 CKD patients undergoing hemodialysis (CKD5-HD) (Hemodialysis Outpatient Clinic) at HUCA and 6 HC were independently recruited as a replication cohort (Supplementary Table 1). Predominant CKD etiology in PD and HD patients were glomerulonephritis (36.3% and 37.5%) and vascular causes (16.1% and 12.5%), with unknown etiology in 22 and 12.5% of patients. None of the CKD patients had a previous diagnosis of diabetes. Exclusion criteria were (i) ongoing immunosuppressive treatment, (ii) concomitant immune-mediated disease or cancer diagnosis, (iii) recent or current infection, (iv) previous CV disease, abdominal aneurism or intermittent claudication, (v) previous carotid surgery, (vi) pregnancy or (vii) diabetes mellitus. Vascular calcifications (VC) were measured by Kauppila scores^45^.

Blood samples were obtained from all study subjects by venipuncture. Automated serum biochemical parameters, lipid analysis and complete blood counts were immediately conducted on all the participants at the Laboratorio de Medicina (HUCA) using routine methods. For additional tests, serum samples were stored at ^−^80 °C. Peripheral blood samples were immediately processed and peripheral blood mononuclear cells (PBMCs) fractions were obtained by centrifugation (1900 rpm, 20 minutes) on density gradients (Lymphosep, Lymphocyte Separation Medium, Biowest, Germany).

### Flow cytometry

PBMCs were treated with FcR Blocking Reagent (Milteny Biotech, Germany) for 20 minutes at 4 °C to avoid unspecific antibody binding to Fc receptors. Then, cells were incubated with CD14 FITC (Immunostep, Spain), CD15 PE-Cy7 (Milteny Biotech), CD16 APC-Cy7 (BioLegend, Germany) and HLA-DR PE (BD Biosciences) or corresponding isotype antibodies for 30 minutes at 4 °C. Cells were then washed with PBS and analyzed in a FACS Canto II flow cytometer (BD Biosciences) equipped with a FACS Diva 6.5 software. First, a ‘live gate’ including all cells subsets, and excluding debris and no cellular events, was designed. LDGs were first gated by their FSC/SSC properties and then, the CD15^+^ population was selected (total LDGs). The percentage (frequency) of each population was computed and referred to the ‘live gate’. Siglec8 expression was analyzed to confirm that eosinophils were not present within CD15^+^ cells, after incubating with a Siglec8 FITC (BioLegend) antibody.

For LDG phenotyping, PBMCs cells were stained as previously explained together with antibodies to granulocyte lineage-specific markers: CD11b PE (BioLegend), CD10 FITC (BioLegend), CD35 FITC (BioLegend), CD66b APC (BioLegend), CD31 PE (Immunostep), CD62L PE (BD Biosciences) or IFNAR1 PE (R&D, Belgium). The intracellular CD68 staining (CD68 PE (BioLegend)) was performed on fixed and permeabilized cells (Cytofix/Cytoperm Kit, BD Biosciences) after extracellular staining. Neutrophil phenotyping was performed in parallel for comparison with LDGs subsets. To this end, whole blood was stained following the previous protocol followed by red blood cell lysis using FACS Lysing Solution (BD) for 5 minutes at 4 °C. Expression levels were measured as the Median Fluorescence Intensity (MFI) for each marker.

### Confocal experiments

CD15^+^ cells were enriched from PBMCs samples by immunomagnetic separation using MojoSort Streptavidin Nanobeads (BioLegend) after incubating with a biotin-conjugated anti-human CD15 antibody (BioLegend) following the protocol provided by the manufacturer. Enrichment yield after sorting was confirmed by flow cytometry. Then, enriched CD15^+^ fractions were seeded in poly-L-lysine coverslips and incubated at 37 °C, 5% CO_2_ for 30 minutes. Cells were washed twice with PBS and incubated with different antibodies [CD16 APC (Immunostep), CD15-biotin (BioLegend) and CD14 FITC (Immunostep) or CD10 FITC (BioLegend)] for 1 hour at 4 °C. Parallel incubations were performed with paired isotypes for each staining. Next, coverslips were washed twice with PBS and cells were incubated with streptavidin-conjugated PE (Immunostep) for 30 minutes at 4 °C. Coverslips were washed twice and cells were fixed with a 4% paraformaldehyde solution for 5 minutes at 4 °C. Finally, coverslips were mounted with Fluoroshield Mounting Medium With DAPI (abcam) and immediately analyzed using a Leica TCS-SP8X Confocal Microscope (Leica Microsystems) using the 63X objective. Images were acquired with LAS X software (Leica), which was also used to produce image overlays. To evaluate nuclear morphology, z-stacks were obtained from 4 μm sections and maximum projections were derived.

### Gene expression analyses

Total RNA from circulating PBMCs was extracted using TRI reagent (Sigma-Aldrich). After reverse transcription using a High-Capacity cDNA Reverse Transcription Kit (Applied Biosystems), quantitative-real time PCR (qPCR) reactions were performed in triplicate using the Stratagene Mx3005P QPCR System (Agilent Technologies), Fast Start Universal Probe Master (Roche) and pre-developed assays (Thermo-Fisher Scientific). Quantification of human DEF3 and CD10 target genes relative to GAPDH expression was performed by comparing threshold cycles using the ΔΔCT method.

### Cytokine quantification

Serum levels of IL-10, IL-6, IL-2, TNFα and IFNγ were measured with a bead-based multiplex assay (BiolegendPlex, BioLegend) analyzed in a FACS Canto II flow cytometer (BD Biosciences) under FACS Diva 6.5, following the protocol provided by the manufacturer. The detection limits were 1.2 pg/ml (IL-10 and IL-2) or 2.4 pg/ml (IL-6, TNFα and IFNγ).

### Statistical analysis

Continuous variables were expressed as median (interquartile range) or mean ± standard deviation, whereas n (%) was used for categorical ones. Differences among groups were assessed by Mann Withney U or Kruskal-Wallis (with Dunn-Bonferroni correction for multiple comparisons) tests, as appropriate. Correlations were assessed by Spearman ranks’ test. Paired tests were used to evaluate differences between subsets from the same sample. Receiver Operating Characteristic (ROC) curves were used to evaluate the adequacy of LDGs subsets as biomarkers, and areas under the curve (AUC) with 95% confidence intervals (CI) and p-values were calculated. To evaluate the additional value of LDGs as biomarkers to conventional variables, z-scores were first derived from the different variables (age and time on dialysis) and combined indices were obtained by summing the individual variables. Multiple regression analyses were conducted to analyze the role of LDGs as a biomarker after adjusting for potential confounders. A p-value < 0.050 was considered as statistically significant. Statistical analyses were performed with SPSS 24.0 and GraphPad Prism 5.0 for Windows.

## Supplementary information

Supplementary Figure 1

## Data Availability

No datasets were generated or analyzed during the current study.

## References

[1] Stenvinkel P, Larsson TE (2013). Chronic kidney disease: a clinical model of premature aging. Am. J. Kidney Dis..

[2] de Jager DJ (2009). Cardiovascular and noncardiovascular mortality among patients starting dialysis. JAMA.

[3] Cannata-Andía JB, Rodríguez-García M, Carrillo-López N, Naves-Díaz M, Díaz-López B (2006). Vascular calcifications: pathogenesis, management, and impact on clinical outcomes. J. Am. Soc. Nephrol..

[4] Rodríguez-García M (2009). Vascular calcifications, vertebral fractures and mortality in haemodialysis patients. Nephrol. Dial. Transplant.

[5] Bessueille L, Magne D (2015). Inflammation: a culprit for vascular calcification in atherosclerosis and diabetes. Cell. Mol. Life Sci..

[6] Benz K, Hilgers K-F, Daniel C, Amann K (2018). Vascular Calcification in Chronic Kidney Disease: The Role of Inflammation. Int. J. Nephrol..

[7] Kochi M, Kohagura K, Shiohira Y, Iseki K, Ohya Y (2018). Chronic kidney disease, inflammation, and cardiovascular disease risk in rheumatoid arthritis. J. Cardiol..

[8] Libby P (2018). Inflammation, Immunity, and Infection in Atherothrombosis: JACC Review Topic of the Week. J. Am. Coll. Cardiol..

[9] Vervloet M, Cozzolino M (2017). Vascular calcification in chronic kidney disease: different bricks in the wall?. Kidney Int..

[10] Beyrau M, Bodkin JV, Nourshargh S (2012). Neutrophil heterogeneity in health and disease: a revitalized avenue in inflammation and immunity. Open Biol..

[11] Deniset JF, Kubes P (2018). Neutrophil heterogeneity: Bona fide subsets or polarization states?. J. Leukoc. Biol..

[12] Mócsai A (2013). Diverse novel functions of neutrophils in immunity, inflammation, and beyond. J. Exp. Med..

[13] Mayadas TN, Cullere X, Lowell CA (2014). The multifaceted functions of neutrophils. Annu. Rev. Pathol..

[14] Hacbarth E, Kajdacsy-Balla A (1986). Low density neutrophils in patients with systemic lupus erythematosus, rheumatoid arthritis, and acute rheumatic fever. Arthritis Rheum..

[15] Cloke T, Munder M, Taylor G, Müller I, Kropf P (2012). Characterization of a Novel Population of Low-Density Granulocytes Associated with Disease Severity in HIV-1 Infection. PLoS One.

[16] Carmona-Rivera C, Kaplan MJ (2013). Low-density granulocytes: a distinct class of neutrophils in systemic autoimmunity. Semin. Immunopathol..

[17] Scapini P, Marini O, Tecchio C, Cassatella MA (2016). Human neutrophils in the saga of cellular heterogeneity: insights and open questions. Immunol. Rev..

[18] Denny MF (2010). A distinct subset of proinflammatory neutrophils isolated from patients with systemic lupus erythematosus induces vascular damage and synthesizes type I IFNs. J. Immunol..

[19] Bennett L (2003). Interferon and Granulopoiesis Signatures in Systemic Lupus Erythematosus Blood. J. Exp. Med..

[20] Dumitru CA, Moses K, Trellakis S, Lang S, Brandau S (2012). Neutrophils and granulocytic myeloid-derived suppressor cells: immunophenotyping, cell biology and clinical relevance in human oncology. Cancer Immunol. Immunother..

[21] Pillay J, Tak T, Kamp VM, Koenderman L (2013). Immune suppression by neutrophils and granulocytic myeloid-derived suppressor cells: similarities and differences. Cell. Mol. Life Sci..

[22] Elghetany MT (2002). Surface antigen changes during normal neutrophilic development: a critical review. Blood Cells. Mol. Dis..

[23] Cossman J, Neckers LM, Leonard WJ, Greene WC (1983). Polymorphonuclear neutrophils express the common acute lymphoblastic leukemia antigen. J. Exp. Med..

[24] McCormack RT, Nelson RD, LeBien TW (1986). Structure/function studies of the common acute lymphoblastic leukemia antigen (CALLA/CD10) expressed on human neutrophils. J. Immunol..

[25] Marini O (2017). Mature CD10+ and immature CD10- neutrophils present in G-CSF-treated donors display opposite effects on T cells. Blood.

[26] Elghetany MT, Ge Y, Patel J, Martinez J, Uhrova H (2004). Flow cytometric study of neutrophilic granulopoiesis in normal bone marrow using an expanded panel of antibodies: correlation with morphologic assessments. J. Clin. Lab. Anal..

[27] Borregaard N, Cowland JB (1997). Granules of the human neutrophilic polymorphonuclear leukocyte. Blood.

[28] Cowland JB, Borregaard N (1999). The individual regulation of granule protein mRNA levels during neutrophil maturation explains the heterogeneity of neutrophil granules. J. Leukoc. Biol..

[29] Rice WG (1987). Defensin-rich dense granules of human neutrophils. Blood.

[30] Cowland JB, Borregaard N (2016). Granulopoiesis and granules of human neutrophils. Immunol. Rev..

[31] Borregaard N, Sørensen OE, Theilgaard-Mönch K (2007). Neutrophil granules: a library of innate immunity proteins. Trends Immunol..

[32] Sagiv JY (2015). Phenotypic diversity and plasticity in circulating neutrophil subpopulations in cancer. Cell Rep..

[33] Villanueva E (2011). Netting neutrophils induce endothelial damage, infiltrate tissues, and expose immunostimulatory molecules in systemic lupus erythematosus. J. Immunol..

[34] Lin AM (2011). Mast cells and neutrophils release IL-17 through extracellular trap formation in psoriasis. J. Immunol..

[35] Singh N (2014). Genomic alterations in abnormal neutrophils isolated from adult patients with systemic lupus erythematosus. Arthritis Res. Ther..

[36] Silverstein DM (2009). Inflammation in chronic kidney disease: role in the progression of renal and cardiovascular disease. Pediatr. Nephrol..

[37] Lam CWK (2009). 2. Inflammation, Cytokines and Chemokines in Chronic Kidney Disease. EJIFCC.

[38] Mihai S (2018). Inflammation-Related Mechanisms in Chronic Kidney Disease Prediction, Progression, and Outcome. J. Immunol. Res..

[39] Carmona-Rivera C, Zhao W, Yalavarthi S, Kaplan MJ (2015). Neutrophil extracellular traps induce endothelial dysfunction in systemic lupus erythematosus through the activation of matrix metalloproteinase-2. Ann. Rheum. Dis..

[40] Grüneboom A (2019). A network of trans-cortical capillaries as mainstay for blood circulation in long bones. Nat. Metab..

[41] López-Bermejo A (2007). Alpha defensins 1, 2, and 3: potential roles in dyslipidemia and vascular dysfunction in humans. Arterioscler. Thromb. Vasc. Biol..

[42] Maneerat Y, Prasongsukarn K, Benjathummarak S, Dechkhajorn W, Chaisri U (2016). Increased alpha-defensin expression is associated with risk of coronary heart disease: a feasible predictive inflammatory biomarker of coronary heart disease in hyperlipidemia patients. Lipids Health Dis..

[43] Joseph G (2008). Plasma alpha-defensin is associated with cardiovascular morbidity and mortality in type 1 diabetic patients. J. Clin. Endocrinol. Metab..

[44] Aragonès G (2016). Proteomic Profile of Unstable Atheroma Plaque: Increased Neutrophil Defensin 1, Clusterin, and Apolipoprotein E Levels in Carotid Secretome. J. Proteome Res..

[45] Kauppila LI (1997). New indices to classify location, severity and progression of calcific lesions in the abdominal aorta: a 25-year follow-up study. Atherosclerosis.

