# Supplementary material for: A subset of low density granulocytes is associated with vascular calcification in chronic kidney disease patients
Source: Sci Rep. 2019 Sep 13;9:13230. doi: 10.1038/s41598-019-49429-x (PMC6744494; doi:10.1038/s41598-019-49429-x)
Supplement: Supplementary file 1 — Supplementary Figure 1 [file 41598_2019_49429_MOESM1_ESM.pdf]

A subset of low density granulocytes is associated with vascular calcification in chronic kidney disease patients

Javier Rodríguez-Carrio, PhD[1,2*]; Natalia Carrillo-López, PhD[2,3*]; Catalina Ulloa, MD[4]; Mariana Seijo, MD[2,5]; Minerva Rodríguez-García, MD, PhD[3,4]; Carmen Rodríguez-Suárez, MD[4]; Carmen Díaz-Corte, MD, PhD[3,4]; Jorge B. Cannata-Andía, MD, PhD[2,3,6#]; Ana Suárez, PhD [1#] and Adriana S. Dusso, PhD[2,3#]

[1] Area of Immunology, Department of Functional Biology, University of Oviedo, Oviedo, Spain.
[2] Bone and Mineral Research Unit, Hospital Universitario Central de Asturias, Instituto de Investigación Sanitaria del Principado de Asturias (ISPA), Oviedo, Spain.
[3] REDinREN-ISCIII.
[4] Division of Nephrology, Hospital Universitario Central de Asturias, Oviedo, Spain.
[5] Laboratorio de Enfermedades Metabólicas Óseas, Hospital de Clínicas, Instituto de Inmunología, Genética y Metabolismo (INIGEM) CONICET- UBA, Argentina
[6] Department of Medicine, University of Oviedo, Oviedo, Spain.

* Equal contribution as first authors

[#] Equal contribution as senior authors

Supplementary Table 1: Demographical, clinical and immunological characteristics of individuals from the replication cohort

	HC (n=6)	CKD5-HD patients (n=16)	p-value
Age, years, mean (range)	57.00 (49.00 – 67.00)	54.87 (21.00 – 74.00)	0.589
Gender, f/m	2/4	6/10	0.856
Clinical features			
Albumin, mg/dl	44.08±2.07	36.08±6.78	<0.001
Urea, mg/dl	38.16±7.08	100.93±29.95	<0.001
Creatinine, mg/dl	0.99±0.20	8.19±2.26	<0.001
Plasma Ca, mmol/l	2.47±0.08	2.19±0.22	0.032
Plasma phosphate, mmol/l	1.01±0.13	1.49±0.60	0.015
PTH, pg/ml	50.20±15.67	379.31±321.74	<0.001
25(OD)-vitamin D, ng/ml	29.72±8.59	8.32±5.85	<0.001
CRP, mg/dl		0.38±0.36	
Vascular calcifications, n(%)		10 (62.5)	
Kauppila score		6.33±4.98	
Time on dialysis, months		31.00 (54.50)	
Treatments, n(%)			
Paricalcitol		7 (43.7)	
Phosphate binders		11 (68.7)	
Statins		9 (56.2)	
Metilprednisone		4 (25.0)	
Epo		13 (81.2)	
Serum cytokines, pg/ml			
IL-2	1.35 (1.05)	1.62 (0.59)	0.876
IL-6	5.12 (3.75)	5.10 (3.04)	0.978
IL-10	1.72 (1.07)	1.50 (1.29)	0.568
IFNγ	8.19 (7.10)	13.14 (8.56)	0.123
TNFα	7.79 (5.35)	10.11 (4.28)	0.453

Supplementary Figure 1: LDG subsets in the replication cohort of CKD5-HD patients. (A) The frequencies of total CD15+ LDGs as well as CD14-CD6-CD15+ and CD14lowCD16posCD15+ subsets in PBMC fractions were compared between HC (open circles) and CKD5-HD patients (black circles). Central bars represent the median values whereas whiskers represent 25th and 75th percentiles. Differences between groups were assessed by Mann-Withney U tests. Association of the LDG subsets in the replication cohort with Kauppila scores (B) and DEF3 gene expression in PBMC fractions (C). Correlations were assessed by Spearman's rank test. (D) DEF3 expression levels in HC and CKD5-HD patients. Differences between groups were assessed by Mann-Withney U tests. * p<0.050, ** p<0.010, *** p<0.001

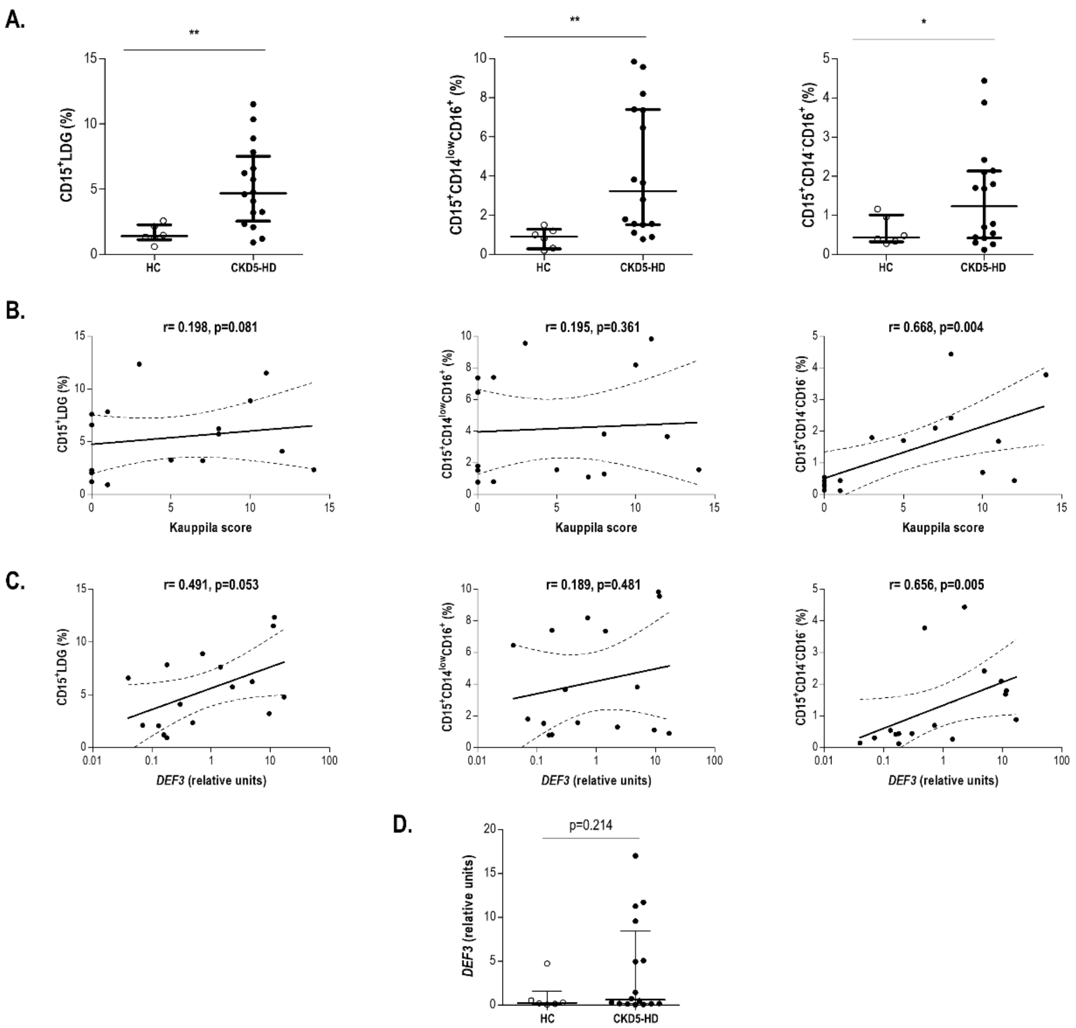

A.
**
*
CD15+LDG (%)
CD15+CD14lowCD16+ (%)
CD15+CD14-CD16+ (%)
HC
CKD5-HD
B.
r= 0.198, p=0.081
r= 0.195, p=0.361
r= 0.668, p=0.004
Kauppila score
C.
r= 0.491, p=0.053
r= 0.189, p=0.481
r= 0.656, p=0.005
DEF3 (relative units)
D.
p=0.214
DEF3 (relative units)
HC
CKD5-HD
